# Recruitment and Succession in a Tropical Benthic Community in Response to *In-Situ* Ocean Acidification

**DOI:** 10.1371/journal.pone.0146707

**Published:** 2016-01-19

**Authors:** Elizabeth Derse Crook, Kristy J. Kroeker, Donald C. Potts, Mario Rebolledo-Vieyra, Laura M. Hernandez-Terrones, Adina Paytan

**Affiliations:** 1 Department of Earth System Science, University of California Irvine, Irvine, California, United States of America; 2 Department of Earth and Planetary Sciences, University of California Santa Cruz, Santa Cruz, California, United States of America; 3 Department of Ecology and Evolutionary Biology, University of California Santa Cruz, Santa Cruz, California, United States of America; 4 Unidad de Ciencias del Agua (UCIA), Centro de Investigación Científica de Yucatán, A.C., Cancún, Quintana Roo, México; Pennsylvania State University, UNITED STATES

## Abstract

Ocean acidification is a pervasive threat to coral reef ecosystems, and our understanding of the ecological processes driving patterns in tropical benthic community development in conditions of acidification is limited. We deployed limestone recruitment tiles in low aragonite saturation (Ω_arag_) waters during an *in-situ* field experiment at Puerto Morelos, Mexico, and compared them to tiles placed in control zones over a 14-month investigation. The early stages of succession showed relatively little difference in coverage of calcifying organisms between the low Ω_arag_ and control zones. However, after 14 months of development, tiles from the low Ω_arag_ zones had up to 70% less cover of calcifying organisms coincident with 42% more fleshy algae than the controls. The percent cover of biofilm and turf algae was also significantly greater in the low Ω_arag_ zones, while the number of key grazing taxa remained constant. We hypothesize that fleshy algae have a competitive edge over the primary calcified space holders, coralline algae, and that acidification leads to altered competitive dynamics between various taxa. We suggest that as acidification impacts reefs in the future, there will be a shift in community assemblages away from upright and crustose coralline algae toward more fleshy algae and turf, established in the early stages of succession.

## Introduction

Declining surface ocean pH (ocean acidification) is a global environmental issue likely to be deleterious for a wide range of marine organisms [1–3]. Coral reef systems are expected to be particularly susceptible to ocean acidification and may see significant declines in calcification over the 21^st^ century due to declining aragonite saturation state (Ω_arag_) [4–6]. Laboratory studies have described responses of many individual species to acidification [7–9], but ecosystem responses to acidification are complex [10,11]. Field studies are essential for understanding how complex assemblages of species may respond to declining pH [12–19].

Ocean acidification is predicted to directly impact calcifying organisms by reducing calcification rates. However, a key question for ecosystems is how acidification may impact communities by altering competitive interactions between organisms, resulting in phase shifts [16, 20–22]. Recently, Kroeker *et al*. at (2012) found that calcareous species were rapidly outcompeted by fleshy algae in acidified conditions; that is, competitive interactions between fleshy algae and calcifying species alter community structure in reduced pH environments in the temperate Mediterranean Sea [16]. Growing evidence suggests that non-calcareous algae appear to benefit in low pH conditions [16, 23–24], while calcifying organisms are either directly impacted (*i*.*e*. reduced calcification rates) or outcompeted by fleshy algal species [14– 16]. However, most studies have been observation-based, and our understanding of the processes driving these patterns in community development is limited. Here, we conducted a field experiment to investigate recruitment and early succession near a tropical coral reef to study how competitive interactions drive ecosystem responses to acidification in a tropical community. We investigate how acidification affects dominance of space among various taxa between sites that vary naturally in carbonate chemistry on the Mesoamerican Barrier Reef. Our study design is similar to that of Kroeker *et al*., (2012), allowing for comparison between the responses in temperate and tropical systems. We focused on interactions among organisms under lower than ambient Ω_arag_ conditions to determine whether, and how, reduced Ω_arag_ may affect this community.

The Mesoamerican Barrier Reef lies off the east coast of the Yucatan Peninsula. Rainwater rapidly infiltrates the porous karstic limestone of Quintana Roo, and then flows towards the ocean through interconnected caves and fractures. Along the flow path, the groundwater mixes extensively with seawater in underground aquifers before discharging into the lagoon between the shore and the offshore reefs at localized submarine springs (known locally as “ojos”) [25]. Although these submarine groundwater springs have near-oceanic salinities and temperatures [26] the water has high dissolved inorganic carbon (C_T_), high total alkalinity (*A*_T_), low pH and low Ω_arag_ [26]. The ojos are typically at 4–7 m depth, and the chemistry of the water affects the diversity, abundance, and calcification rates of corals that settle and grow at the springs [18,27].

Understanding the ecological processes leading to the observed differences in diversity and abundance of organisms along these naturally varying pH-saturation conditions is a critical step for predicting future impacts of acidification on reef environments. We deployed limestone recruitment tiles in low pH-Ω_arag_ waters at the ojo centers and compared them to those concurrently placed in control zones of ambient pH-saturation within a few meters of the springs. A subset of tiles was collected on three occasions (3 months, 6 months and 14 months) for analysis of recruitment and community succession. Although the average saturation state at the ojo centers (Ω_arag_ = 1.5) is much lower than most predictions for the late 21^st^ century, this study assesses potential impacts of ocean acidification on developing reef communities that may be particularly relevant if atmospheric CO_2_ follows more extreme IPCC scenarios [28] or if local conditions (e.g. river or groundwater inputs, upwelling) exacerbate global acidification.

## Materials and Methods

**Fig 1:**
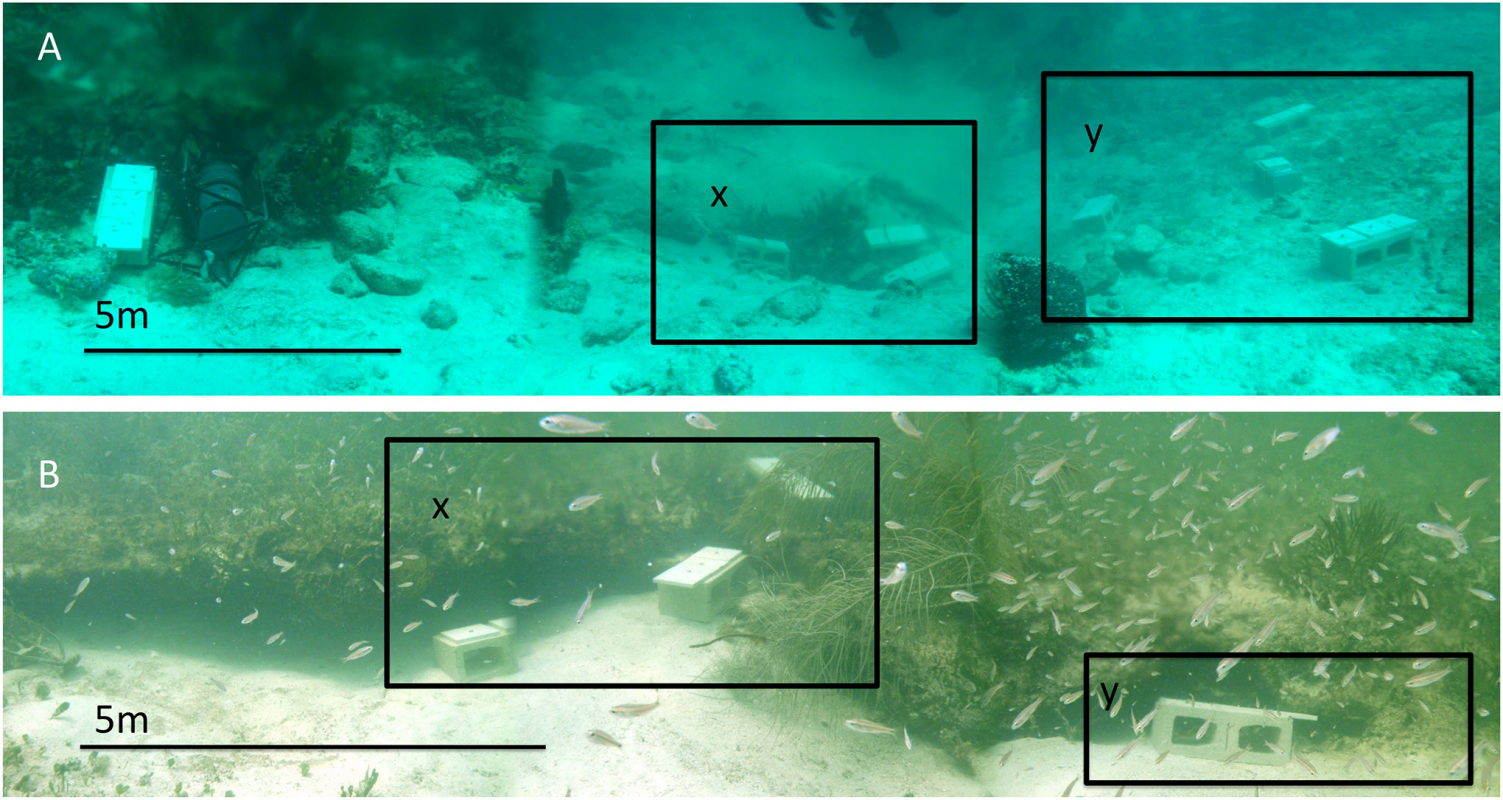
The two ojo sites. Ojo A (A) and Ojo B (B) during recruitment substrate deployment (time zero). The low pH-Ω_arag_ zones (x) are within 10m of ambient zones (y) at each site.

**Fig 2:**
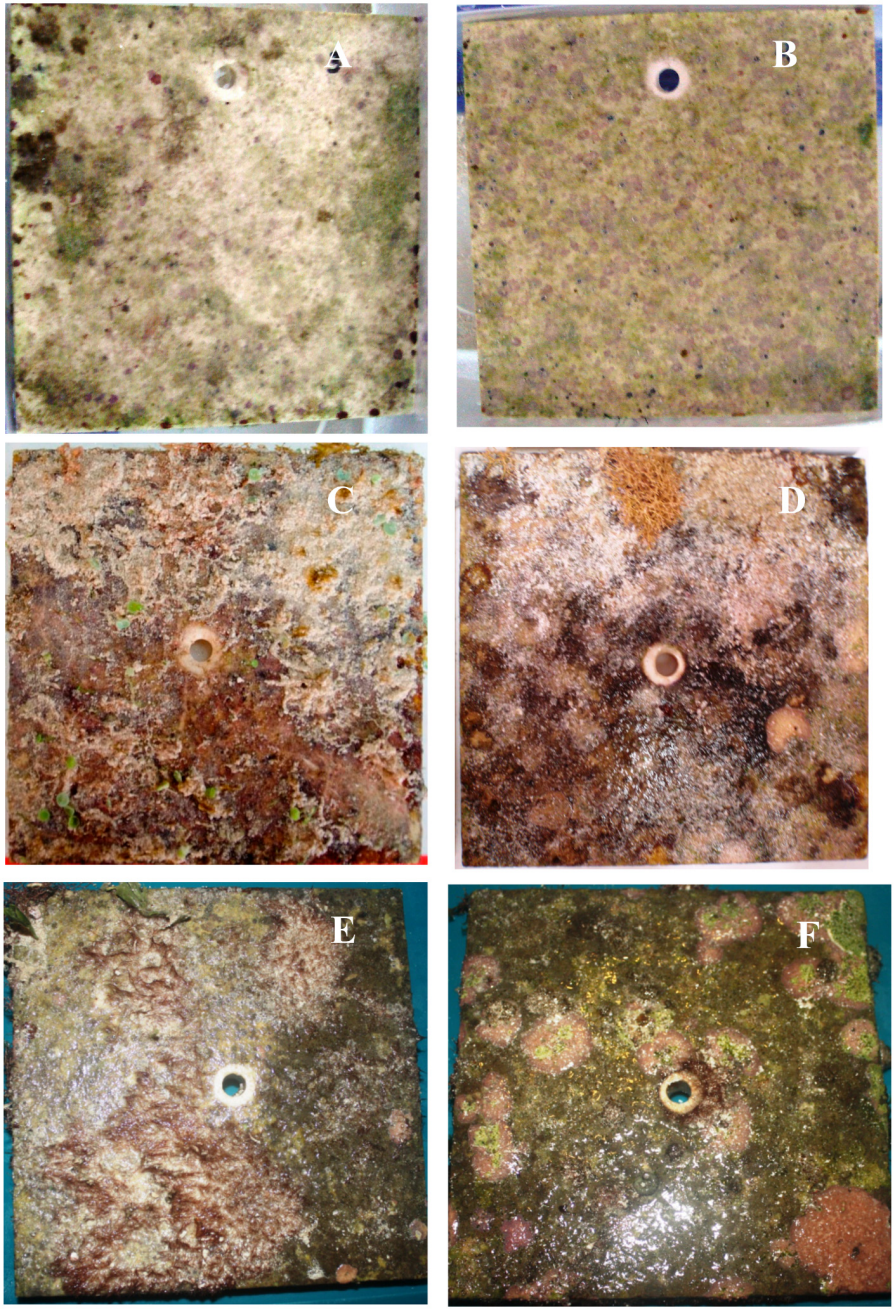
Tiles after recovery. Examples of tiles collected at 3 months (A, B), 6 months (C,D), and 14 months (E,F), at ojo centers (A,C,E) and controls (B,D,F).

The experiment took place at two ojos (Sites A and B), approximately 500 m offshore near Puerto Morelos Reef Natural Park, Quintana Roo, Mexico (20.853° N, 86.898° W). The ojos were chosen based on previous monitoring, which suggested their water had low saturation (Ojo A Ω_arag_ = 1.4 ± 0.4, Ojo B Ω_arag_ = 1.6 ± 0.4) for much of the time each year and relatively high salinities (> 30) in the immediate vicinity of the discharge (Table 1) [26, 27]. We used a 2 x 2 factorial design (2 Ω_arag_ levels x 2 ojo sites), which allowed us to compare the response as a function of chemical changes (e,g. different chemistry regimes with low pH-Ω_arag_ and ambient pH-Ω_arag_) and location (site A and site B). If aragonite saturation state or pH is an important controlling factor we expect little difference between sites at similar pH-Ω_arag_ and larger differences regardless of site for different pH-Ω_arag_. To mimic the natural karst substrate, we deployed 40 limestone tiles (15 x 15 cm), acquired from a quarry near Puerto Morelos. Twenty tiles were deployed at each site; 10 in a low saturation zone (Ω_arag_ ~ 1.5, hereafter referred to as “ojo”) in the direct vicinity of the spring discharge, and 10 in an ambient zone (Ω_arag_ ~ 3.8, hereafter referred to as “control”) about 5 to 10 m from the area of influence of the spring discharge. The tiles were bolted to concrete masonry blocks with stainless steel screws through a hole drilled in the center of each tile (Fig 1). We deployed the tiles on 28 August 2010, immediately preceding a coral mass-spawning event. We removed subsets of three randomly selected tiles from each treatment after 3 months (25 November 2010) and 6 months (14 March 2011), and removed the four remaining tiles after 14 months (19 October 2011). Upon removal, the tiles were photographed, fixed in a 4% formalin solution for 48 hours, and then stored in 70% ethanol until analyzed (Fig 2).

**Table 1:** Water Chemistry at the two ojo sites and controls.

	Ojo A	Ojo B	Control A	Control B
N	67	57	29	26
A_T_ (μmol/kg)	2676 ± 46	2797 ± 65	2380 ± 34	2396 ± 54
C_T_ (μmol/kg)	2631 ± 48	2526 ± 56	2060 ± 57	2089 ± 70
pH_T_	7.60 ± 0.04	7.50 ± 0.07	8.10 ± 0.04	8.09 ± 0.05
Ωarag	2.3 ± 0.2	1.8± 0.2	3.9 ± 0.5	3.6 ± 0.6
Temp (°C)	28.0 ± 0.2	26.9 ± 0.2	27.7 ± 1.6	28.0 ± 1.7
Salinity	32.2 ± 0.5	30.6 ± 0.4	33.36 ± 2.2	33.5 ± 2.3
NO_3_ (μM)	8.0 ± 5.9	9.1 ± 4.4	1.5 ± 0.4	2.83 ± 2.3
PO_4_ (μM)	0.21 ± 0.03	0.25 ± 0.04	0.10 ± 0.03	0.06 ± 0.02
Si (μM)	10.7 ± 1.2	11.6 ± 1.9	2.4 ± 0.5	5.69 ± 1.2

Saturation and pH_T_ were calculated from discrete water samples for dissolved inorganic carbon (C_T_) and total alkalinity (A_T_). Values are averages from N = x samples collected over the 14 months deployment, ± Standard Error.

We collected discrete water samples for dissolved inorganic carbon (C_T_), total alkalinity (A_T_), salinity, and nutrients at initial deployment, during each recovery, and at monthly intervals throughout the 14-month deployment. Additionally, in situ monitoring was conducted intermittently over a 4 year period before, during, and after deployment in an attempt to capture the variability in chemistry at the ojo sites over shorter (e.g., diurnal) and longer (e.g., interannual) time frames. These include a 24 hour monitoring at Ojo A. Values reported here (Table 1) include discrete samples taken over a several year period to give a greater indicator of conditions at the site. C_T_ was measured using a CM5011 Carbon Coulometer (UIC, Inc.) and *A*_T_ was measured with an automated, open-cell potentiometric titration procedure. Certified Reference Materials (batch 118) from the laboratory of Dr. Andrew Dickson at Scripps Institution of Oceanography were used to calibrate each instrument. C_T_ and A_T_ were used to calculate aragonite saturation state (Ω_arag_) and pH via CO_2_sys software [29], using CO_2_ dissociation constants from Merhbach et al. (1973) refitted by Dickson and Millero (1987) [30,31]. pH is reported in total scale (pH_T_). Salinity was measured with a salinometer (Guildline 8410 PortaSal), and nutrients were analyzed on a flow injection autoanalyzer (FIA, Lachat Instruments Model QuickChem 8000). In addition to the discrete samples, pH, temperature, and salinity at the ojos were monitored using a SeapHOx sensor, which suggest that the discharge at the spring was continuous throughout the experiment (S1 Fig).

**Fig 3:**
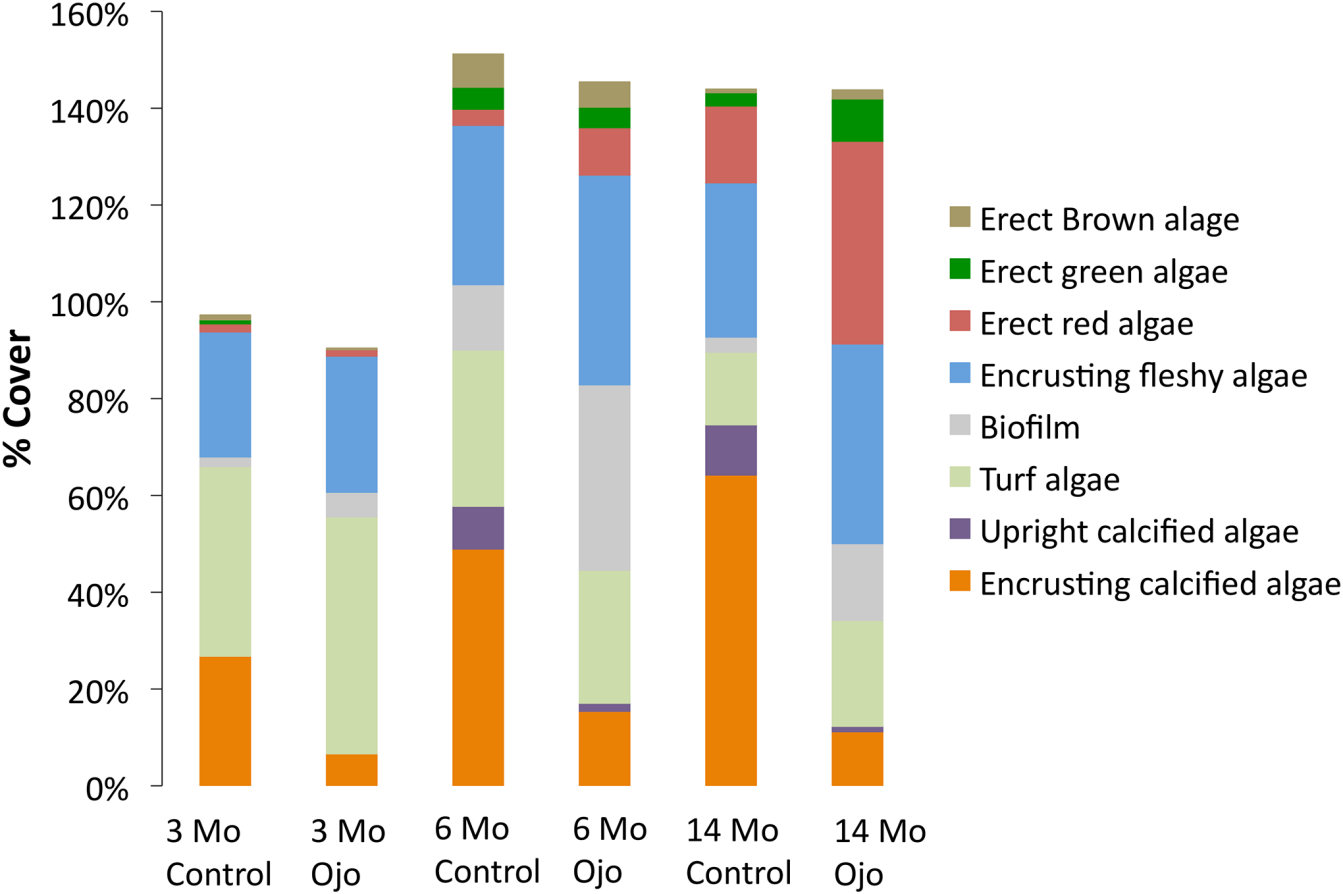
Average percent cover by taxonomic group (Sites A+B) at 3, 6 and 14 months. Percent cover can be greater than 100% due to the multiple layers of organisms present on the tiles. Foraminifera, molluscs, and polychaetes are not represented due to their minor contribution to overall % cover.

The main focus of this study was to determine how acidification may impact community level changes, and specifically, to determine functional differences between the communities inside and outside of the springs. We therefore focus on functional groups rather than conducting species level analyses. This approach is consistent with previous investigations [16] and thus allows comparison between the Caribbean and Mediterranean sites. Organisms on the tiles were assigned to eleven functional groups (Fig 3). The tiles were divided into 1.5 x 3 cm subplots on the edges and 3 x 3 cm subplots on the face of the tiles for visual estimates of percent cover [16]. Subplot estimates were then summed for total percent cover. The cover of erect fleshy algae forming a canopy over the tile was analyzed first and then removed to estimate the percent cover of encrusting groups. Encrusting foraminifera, molluscs, and polychaetes were counted and measured using a Celestron digital microscope (0.1 mm accuracy).

Community composition, defined as the presence or absence of functional groups, was compared between chemistry regime (ojo or control zones) and sites (Site A or B) for the 14-month tiles on a Bray-Curtis (BC) similarity matrix of presence/absence of functional groups. Community structure, defined as the relative abundance of functional groups, was analyzed on a zero-adjusted BC similarity matrix of square-root transformed total percent cover of functional groups. Permutational Multivariate Analysis of Variance (PERMANOVAs) were used to test variation in community composition and structure, with site and chemistry regime as categorical, fixed factors using 9,999 unrestricted permutations of the transformed data and Type III SS. Non-metric multi-dimensional scaling (nMDS) plots were made to visualize the variability in community structure. NMDS plots are ordinations of the multivariate data, in this case community data, where each point on the ordination represents the community on a single tile. In an nMDS plot, the multivariate data are placed into two-dimensional space so that the rank differences among the data are preserved, based on the BC dissimilarity matrix. Thus communities that are more dissimilar to one another are farther apart on the plot.

Variation in succession was tested on the community structure measurements among *site* x *saturation* x *time* using PERMANOVA with site (Site A or B), chemistry regime (ojo or control zone), and time (3, 6 or 14 months) as fixed factors. In addition, we tested for differences in the percent cover of select fauna. Due to numerous zero values that violated the assumptions of parametric statistics, we used permutation-based analysis of variance to test univariate variables (α = 0.05).

All work was conducted at the Puerto Morelos Reef Natural Park. Samples were collected under Secretaría de Agricultura, Ganadería, Desarrollo Rural, Pesca y Alimentación (SAGARPA) permit DGOPA.00153.170111.-0051 and were exported with a Convention on International Trade in Endangered Species (CITES) Permit MX52912.

## Results

Eleven functional groups were common on the tiles (Figs 2 and 3). Five groups were comprised of calcareous organisms: including erect and crustose coralline algae (CCA), vermetid molluscs, tubicolous polychaetes, and encrusting foraminifera. Six groups were comprised of non-calcareous forms: including red, green and brown erect fleshy algae, turf algae, encrusting fleshy algae, and bacterial biofilm. Because only a total of 4 individual corals settled (*Siderastrea radians*) on tiles, only at the control sites, they were excluded from the analyses. The tiles from the ojos were generally dominated by fleshy algae, turf, and biofilm, while those in control conditions were dominated by CCA (Fig 3). Erect coralline algae were often entirely absent from ojo tiles.

**Fig 4:**
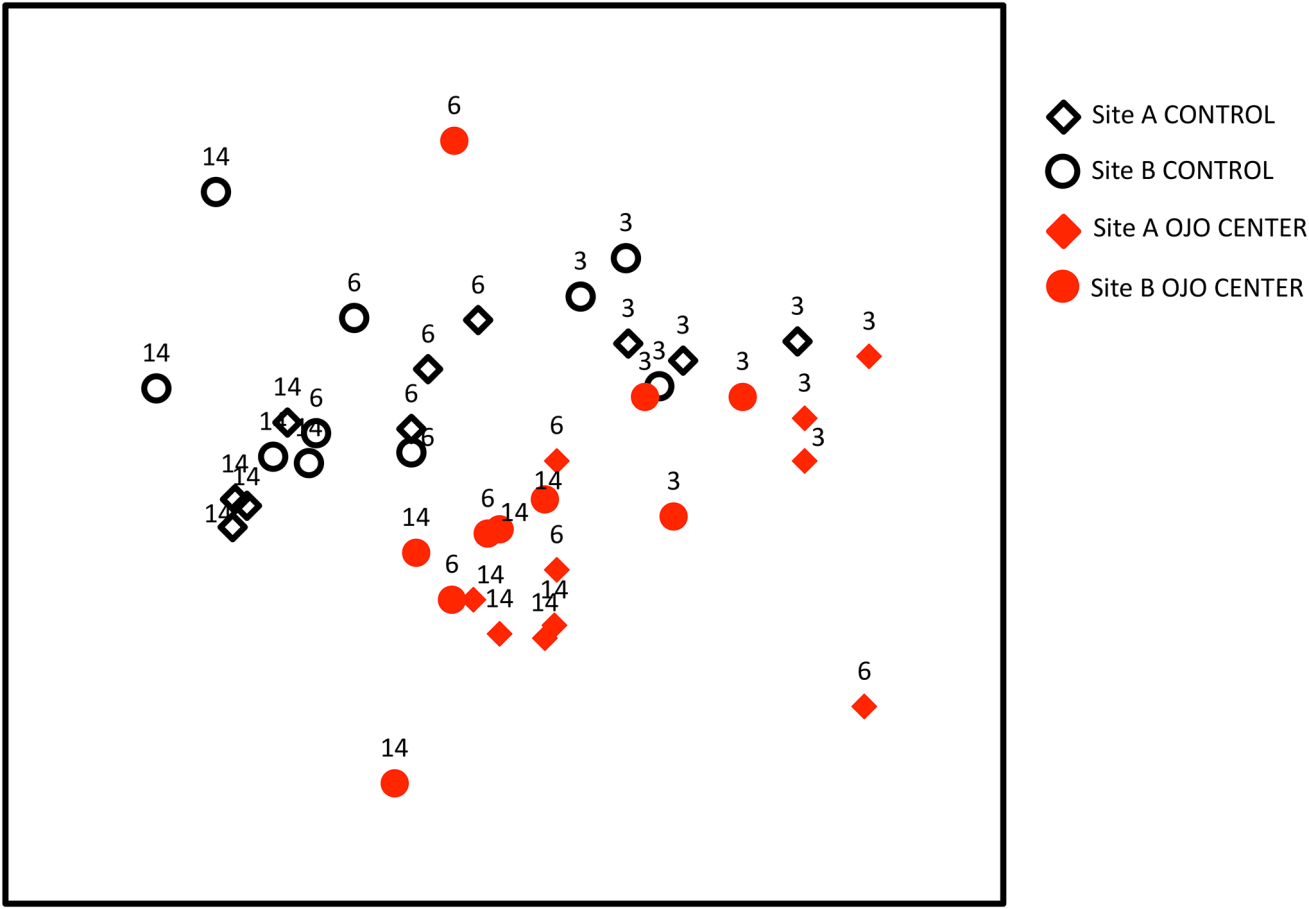
non-metric Multidimensional Scaling (nMDS) for community structure. In an nMDS, distance on the plot is a measure of dissimilarity. Each dot is representative of a single tile, labeled by site (open circles (site A) or closed circles (site B)), time (3, 6, or 14 months) and saturation state (red for ojo centers, black for controls). In an nMDS plot, multivariate data are placed into two-dimensional space so that the rank differences among the data are preserved, based on the BC dissimilarity matrix. Thus communities that are more dissimilar to one another are farther apart on the plot.

**Fig 5:**
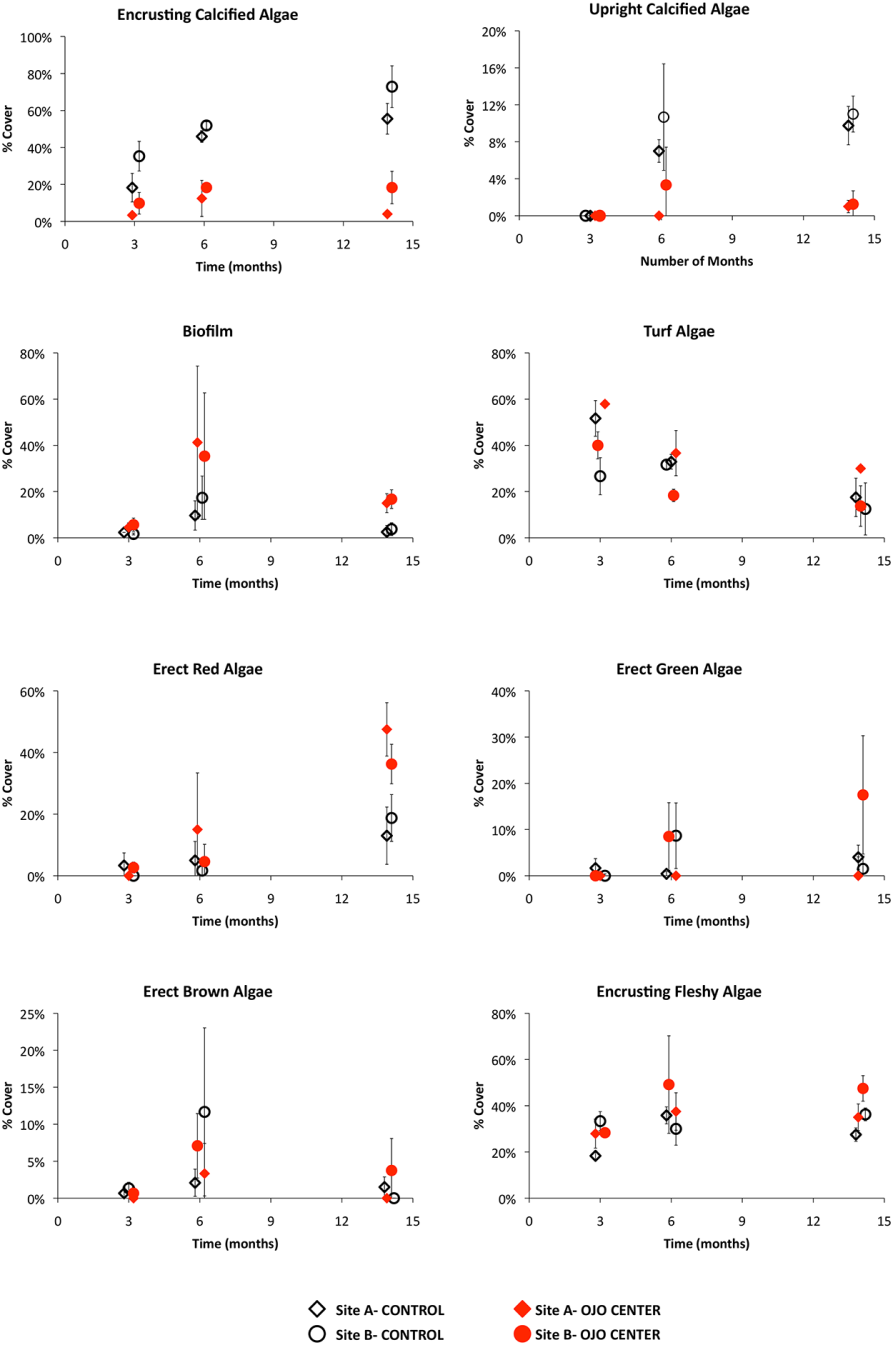
Average % cover of each taxa over time. Open black symbols represent controls and closed red symbols represent the ojo centers. Diamonds depict Site A and circles depict Site B. Error bars are ± S.D. Note that the vertical (y-axis) scales on each figure are different.

After 14 months of development, the community structure, defined as the relative abundance of functional groups, was significantly different between ojo and control conditions (PERMANOVA *chemistry*, F_1,12_ = 14.89, p = 0.0001; Fig 4). The differences in community structure between ojos and controls were driven primarily by higher abundances of CCA and erect calcified algae in the ambient zone, while the ojos had higher abundances of biofilm, erect fleshy algae, and turf algae (Fig 5). After 14 months, there were relatively minor differences between sites A and B (F_1,12_ = 2.96, p = 0.05). There were only marginal differences in the relative abundance between sites, with erect red algae being slightly more abundant at Site A and erect green algae and encrusting calcified algae being slightly more abundant at Site B. However, the site variable contributed only minimally to variation, and the impact of the sites was negligible compared to the impact of Ω_arag_ (Fig 4).

In addition to the differences in the relative abundance of functional groups, the composition of the assemblages, defined as the presence or absence of functional groups, also varied between chemistry regimes (PERMANOVA, F_1,12_ = 7.44, p = 0.002). These differences in community composition were mainly due to the absence of erect and crustose coralline algae (CCA) on some, but not all, of the ojo tiles. In contrast, the community composition did not differ between sites (PERMANOVA *site* F_1,12_ = 0.24, p = 0.76) after 14 months of development.

Community structure changed through time (PERMANOVA *time* F_2,28_ = 17.64, p = 0.0001) in both chemistry regimes. Significant differences between chemistry regimes (F_1,28_ = 18.14, p = 0.0001) and sites (F_1,28_ = 3.54, p = 0.02) were maintained through time. Within each chemistry regime and site, however, the community structure only differed between 3 months and the following time points, but not between 6 and 14 months.

**Fig 6:**
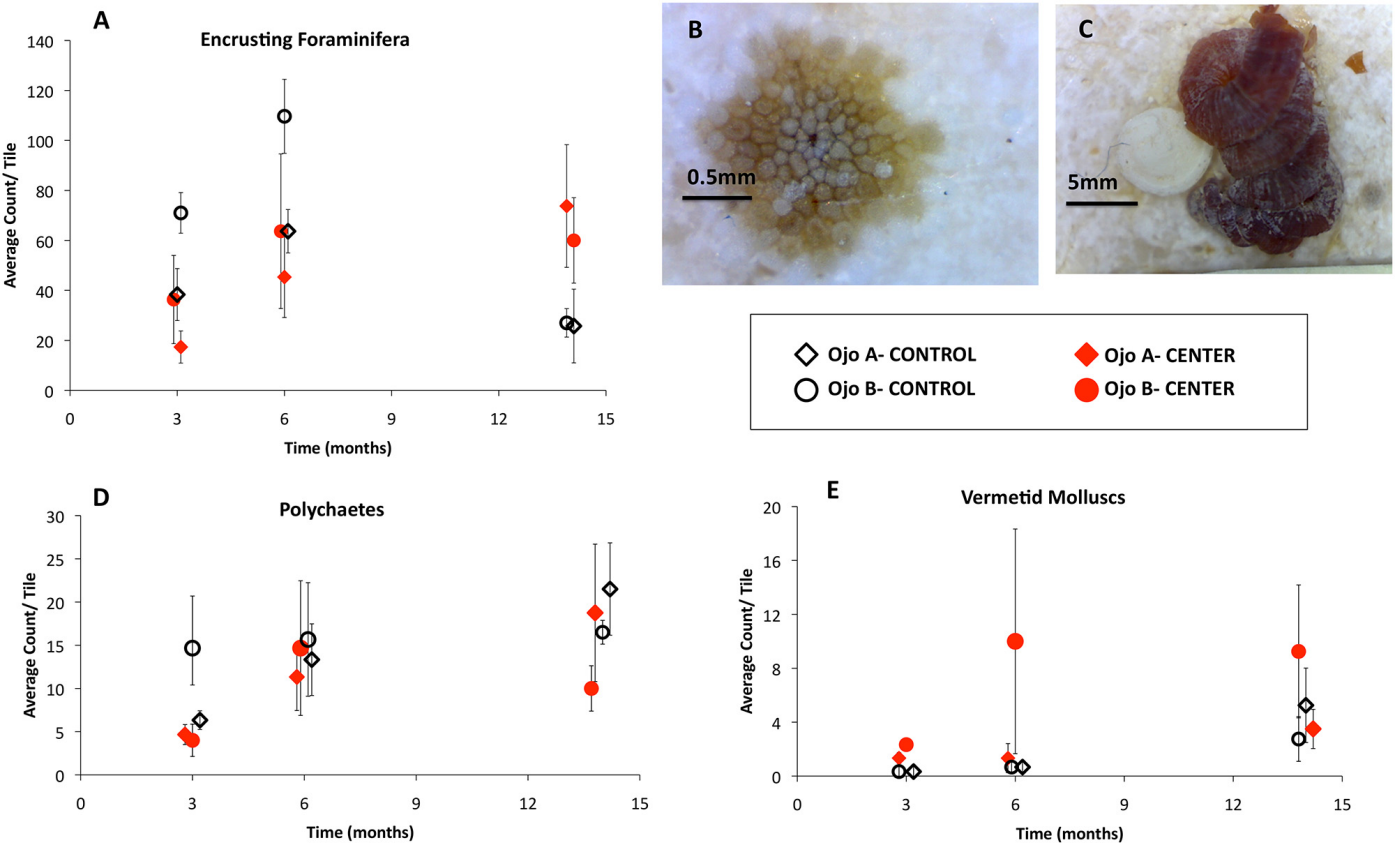
Encrusting foraminifera, polychaete, and vermetid mollusc abundance. (A,D,E) Average encrusting foraminifera, polychaete, and vermetid mollusc abundance (# of individuals) for each set of tiles by month. Open black symbols represent controls and closed red symbols represent the ojo centers. Diamonds depict Site A and circles depict Site B. Error bars are ± S.D. Note that the vertical (y-axis) scales on each figure are different. Visual examples of encrusting foraminifera (B) and vermetid molluscs (C) found on the tiles. Images taken with a 0.1mm accuracy digital microscope.

Foraminifera abundance (Fig 6B) was affected by both chemistry regime and time (ANOVA *pH x time*, F_2,28_ = 6.44, p = 0.005). On control tiles, the number of foraminifera increased from 3 to 6 months (paired t-test, p = 0.01), and then declined by 14 months (p = 0.0007) (Fig 6a). The number of foraminifera was greater on control than ojo tiles at 3 months (p = 0.04), but did not differ at 6 months (p = 0.1). At 14 months, there were more foraminifera at the ojos than at the controls (p = 0.03), stemming from a significant decline in their abundance at the controls. The number of foraminifera at the ojo centers increased marginally from 3 to 14 months (p = 0.06). Conversely, no trends were found in relation to chemistry regime for either polychaete or vermetid molluscs (Fig 6c) abundance, although vermetids increased in number over time (PERMANOVA *time*, F_2,28_ = 5.42, p = 0.009; Fig 6b and 6c).

**Fig 7:**
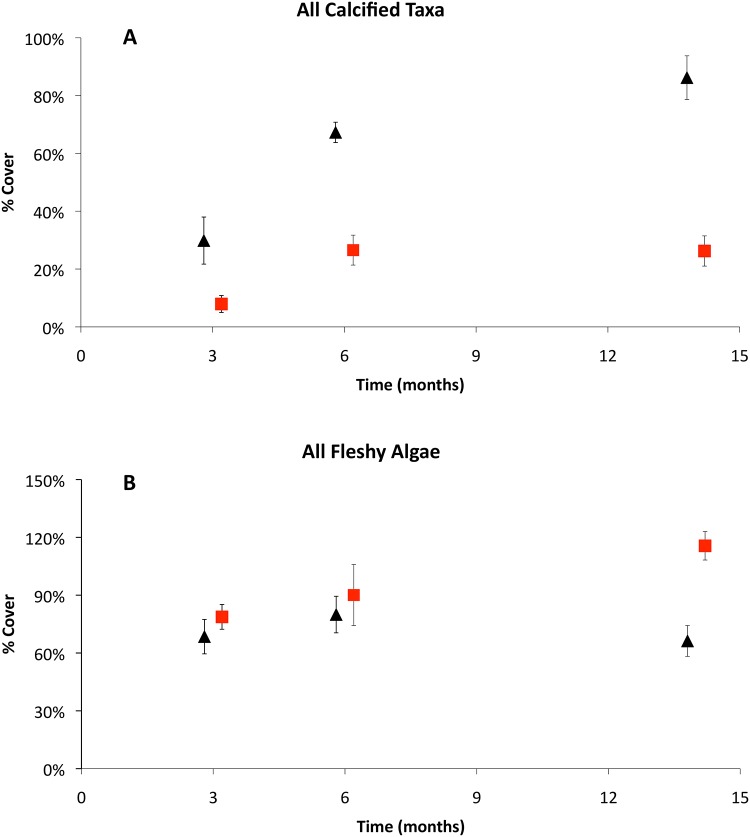
Aggregate community indices. Aggregate calcified taxa (A) and combined fleshy algal indices (B) by month. Each point represents the mean across both sites for Sites A and B. Black triangles are controls and red squares are ojo centers. Error bars are ± S.D.

To address the small relative percent cover of fauna present on the tiles, the percent cover estimates of all calcified taxa (calcareous algae and all animals) were grouped for a univariate comparison. The total percent cover of all calcified taxa was greater in the controls than in the ojos at all time periods (Fig 7a). Importantly, the percent cover of all calcified taxa increased significantly among all time periods on the control tiles, but did not increase substantially between 6 and 14 months on the ojo tiles. Rather, the percent cover of all calcified taxa stagnated on the ojo center tiles at 6 months.

An additional univariate comparison was made for aggregate fleshy algal indices (summing erect green, red, and brown algae and turf algae). Total fleshy algal coverage was significantly greater at the ojo centers than at the controls at each time point, and by 14 months, was 42% greater at the ojos (Fig 7b).

## Discussion

After 14 months of recruitment and development, there were significant differences in species composition and relative abundance of species present on the ojo tiles compared to the controls. Differences in community structure between ojos and controls were primarily due to greater percent cover of erect and crustose coralline algae on the control tiles. In our study, both upright and crustose coralline algae (CCA) were found at the ojos in low saturation conditions, although coverage was significantly reduced compared to control sites. CCA appear to recruit early regardless of saturation, suggesting that these species may be physiologically tolerant to low saturation waters during early settlement and growth. However, the development of the CCA ceased after 3 months at the ojos, and by 14 months, the ojo tiles had 82% less CCA than controls. This finding is similar to laboratory results of Kuffner *et al*. (2007), who estimated a drop in percent cover of CCA of more than 90% for a similar decrease in saturation (Ω_arag_ = 1.5) [32]. On average, erect calcified algae had 89% less cover near the ojos and were conspicuously absent from numerous ojo tiles altogether.

This cessation of development in the percent cover of the coralline algae on the ojo tiles coincided with an increase in percent cover of fleshy algae, a trend that was consistent through time and which resulted in 42% greater coverage by fleshy algae at ojo centers after 14 months of development. While there was variability among the functional groups present on the tiles, the aggregated fleshy algal coverage was greater on ojo tiles compared to controls at each time point. Similar trends were observed by Kroeker *et al*. (2012) at the CO_2_ vent site at Ischia, Italy, in which calcareous species did not increase in percent cover after 6 months, which was attributed to overgrowth or space occupation by fleshy and turf algae. Fleshy and turf algae can limit CCA by overgrowth and subsequent light limitation [33–35] or by occupying the space needed for recruitment. Numerous studies have highlighted the importance of disturbance (e.g., grazing [36], scour [37], or epithelial sloughing [38] in mediating algal overgrowth of CCA, and without these mechanisms, CCA are generally subordinate to fleshy algae. Importantly, we did not find grazers or have high sources of disturbance on our tiles. Our study is consistent with the idea that despite some species of CCA being physiologically capable of recruitment and growth in conditions of low pH-saturation, competitive interactions between fleshy algae and coralline algae at high pCO_2_ could reduce the percent cover of coralline algae [20]. This competitive advantage of fleshy alga over other taxa was also noted by Connell and Russell (2010), who observed that space occupation by fleshy algae increase at high pCO_2_, and Kuffner *et al*. (2007) who concluded that turf algae can limit CCA abundance in high CO_2_ [19,32]. This is in contrast, however to a recent study by Fabricius et al. (2015) at naturally acidified reefs in Papua New Guinea, which found CCA cover decreased with pH despite low cover of fleshy algal species [39]. Differences among these studies could in part be attributed to higher grazing pressure at the PNG site, which could have limited fleshy algal cover at this site, or species-specific differences in response to high CO_2_. Our study implies that other anthropogenic stressors that allow fleshy algae to flourish on coral reefs, such as nutrient loading [40,41] or overfishing [42,43], could exacerbate the effects of ocean acidification on CCA development and cover. That is, fleshy algae appear to have a competitive edge over corallines in high CO_2_ (but see Short et al. 2014) [44], and additional stressors to coralline communities, or any factor that would give more advantage to the fleshy algae (*i*.*e*. higher nutrient levels or decreased herbivory) [40,45,46], could compound the indirect effects of acidification to coralline communities.

It is likely that changes in pCO_2_ may impact a range of physiological processes in both fleshy and coralline algae, with consequences to the community assemblages that can preferentially recruit and grow [47]. There is a critical need to understand how pCO_2_ will impact photosynthesis, enzyme systems, and reproduction rates in various marine algae, and thus predict which species of algae will flourish in future reef environments. Our study lends support to the idea that advantages in carbon transport in fleshy macroalgae are equally as important to understand as carbon transport and calcifiction in coralline algae.

Coralline algae are important ecological components of a coral reef, as they cement the reef framework and provide chemical settlement cues and settlement substrate for coral larvae [48,49]: understanding the response of coralline algae to ocean acidification is therefore of critical importance. A dramatic decline in coverage under conditions of low pH-saturation indicates that both the basic framework of reefs and the recruitment of corals could decrease with ocean acidification. Doropoulos *et al*. (2012) investigated coral recruitment in response to reduced coralline algae abundance at high pCO_2_ and found that coral settlement was mediated by settlement cues from CCA’s, which were most heavily impacted by saturation state [49]. Their study noted a greater than 45% decline in coral recruitment due to declining CCA and loss of settlement cues. In our study, the experimental substrates were deployed in August (a likely time for coral mass-spawning) and retrieved 14 months later in the hopes of capturing at least one mass-spawning event. Despite this, the number of corals that recruited and settled on the tiles was not sufficient to address how acidification may impact coral recruitment and growth. As the recruitment substrates were placed in a lagoon with low overall coral coverage, this was not unexpected. However, the 14-month control tiles had a total of 4 colonies of *Siderastrea radians*, while no corals were present on the low saturation tiles, a trend that we feel is worth noting. The small juvenile colonies present suggested the corals were recent recruits. As previously noted, corals rely on important settlement cues from coralline algae, and as the low saturation tiles had approximately 80% less CCA than the control tiles, they may have been less hospitable to coral larvae. Our study suggests that this potential decline in coral recruitment can in part be attributed to the competitive advantage of fleshy algae over CCA in more acidic conditions. This connection between the impact of fleshy algal coverage, CCA, and coral recruitment warrants further study.

When considering all calcareous species (flora and fauna combined), the difference in percent cover between control and ojo tiles was similar at all time points, with approximately 70% less cover on the ojo tiles. The percent cover at the controls increased over time; however, in the ojo zones, there was no increase in calcifying cover after 6 months. Unlike the Ischia site, where no calcareous species were found at Ω_arag_ = 1.2 [16], low saturation at Puerto Morelos (Ω_arag_≤1.5) still had up to 30% cover of calcifying organisms. This could be because the calcareous species present at Puerto Morelos are naturally more resilient to acidification, or because calcification continues to drop as the saturation level decreases reaching negligible levels at Ω_arag_ = 1.2. Regardless, this finding has significant implications for future reef development, as the reduction in calcified taxa was immediate and persistent throughout the duration of the deployment.

The observed patterns are also consistent with competition between calcifying taxa. The trends observed for encrusting foraminifera (Fig 6) over the 14-month study suggest that CCA have a decreased ability to compete under acidification conditions. While the abundance of encrusting foraminifera increased from 3–6 months by 40% on the control tiles, at 14 months when CCA became established, the trend reversed and they decreased by 70%. Visual analysis of the tiles revealed that many of the foraminifera were overgrown by CCA between 6 months and 14 months, suggesting CCA out-competed the foraminifera for remaining space. This trend was not seen on the ojo tiles. Instead, the number of foraminifera continued to increase on the ojo center tiles where CCA were not able to establish dominance and were 80% less abundant. As with the calcifying algae, our results suggest that the calcifying foraminifera were able to grow in the ojo conditions, and that community changes were primarily driven by competition among species, where those that did not compete with CCA occupied more space on the hard substrate.

Differences in community structure between chemistry regimes were also reflected by a higher abundance of biofilm on the low saturation tiles. By 14 months, biofilm cover was significantly higher on the ojo than at the control tiles, a trend that is also consistent with the Kroeker *et al*. (2012) study. Additionally, in our study, there was a significant increase in the amount of biofilm in both zones at 6 months. Biofilms are essential components of marine ecosystems, as they are food for a number of grazers [50,51], and more importantly to this study, likely mediate the settlement and metamorphosis of benthic organisms [52–55]. Biofilms begin to settle on substrates within hours of submersion and are associated with early stages of succession [51,52]. The significantly higher percent cover of biofilm on the ojo tiles after 14 months of development could suggest they remained in an earlier stage of development for a longer time compared to the control tiles (as seen at Ischia). The increase in biofilm on both tiles at 6 months of growth can potentially be explained by seasonal variability (the tiles were removed in the winter months with lower incident radiation), as biofilm abundance has been shown to be inversely related to solar stress [50,51].

There were no obvious trends in vermetid mollusk or polychaete abundance by site or zone. In fact, the vermetid mollusk abundance increased significantly by 14 months on the control tiles. This observation of resilience by certain taxa to acidification is mirrored by several studies to date [8,16,17,56,57]. However, as these organisms were likely only exposed to low saturation conditions after settlement on the tiles, it is not clear from our study if acidification may impact earlier life history stages. It is possible that these taxa are impacted more in the early larval stages before settlement, and that “carry-over” effects which are expressed only in adults that were exposed to acidification during early stages of growth [58] are responsible for the discrepancy seen between the populations in this study and those of laboratory experiments.

While *in-situ* field studies are valuable for investigating how complex assemblages may respond to acidification, multiple environmental parameters may co-vary, making it difficult to resolve the influence of Ω_arag_ on the community assemblages from that of other factors, or to assess the extent to which the influence of Ω_arag_ may be modulated by other, co-varying factors. While temperature and light are comparable between the ojo and control zones in our study, salinity and nutrients often co-vary with changes in saturation state in the ojos as they are all dependent on the flux of submarine groundwater discharge. It is therefore important to compare our results to those derived from additional field studies where Ω_arag_ conditions are not coupled with groundwater discharge. At the Ischia volcanic vent site, there were no salinity or nutrient changes associated with pH zones, and the similarity of our results with those of Kroeker et al. (2012) (after which this investigation was designed) lends weight to the idea that the carbonate chemistry is the primary driver of change in the communities at the ojos. Specifically, the similarities in community structure and composition with respect to CCA and fleshy algae suggests these changes may be due to low pH-saturation exposure.

Our study illustrates that while acidification will have significant direct impacts on calcification, the altered competitive interactions between organisms will also impact community assemblages in the future. That is, we expect to see a shift in communities from coralline algal coverage to fleshy algae over time as pCO_2_ increases over the 21^st^ century. It is important to note that the tropical benthic calcifying organisms were able to recruit and grow in low Ω_arag_ conditions. However, we hypothesize that competition for space as the community developed was the leading driver in the community shifts observed. Our study illustrates the importance of observing the response of entire communities to OA, as interactions between organisms will compound the direct effects of acidification and likely increase reef degradation beyond the estimates derived from species-specific observational studies. This study thus illustrates the need for conservation and policy decisions that will consider community-wide responses to acidification, particularly with regard to the increased competition between calcifying and fleshy algal species at decreased saturation. For instance, if fleshy algal species are more successful at high nutrient levels, then mitigation strategies that reduce eutrophication of surface waters may help fleshy algae from having an even greater competitive edge over calcifying species. As the oceans become more acidic over the 21^st^ century, it will become essential to alleviate human impacts that have the potential to compound competitive interactions between organisms.

## Supporting Information

S1 Fig Salinity and pH over time as measured by an autonomous sensor. Salinity and pH were measured at 15 minute time intervals for a period of 3 months (August-October 2010) for a total of over 5500 data points at a single spring. Salinity is plotted against pH (**a**), and grouped according to the number of data points occurring in a given salinity range (**b**). As depicted, 93% of data points fall above a salinity of 30, and salinity never drops below 27 at the center of discharge. The lower salinity conditions are during low tide in the rainy season and the conditions do not prevail for more than a one hour. Agreement (1 s.d.) between the pH of the sensor values and the discrete measurements (calculated pH) is approximately ±0.07.
(DOC)
