# Supplementary material for: Recruitment and Succession in a Tropical Benthic Community in Response to In-Situ Ocean Acidification
Source: PLoS One. 2016 Jan 19;11(1):e0146707. doi: 10.1371/journal.pone.0146707 (PMC4718464; doi:10.1371/journal.pone.0146707)
Supplement: S1 Fig — Salinity and pH were measured at 15 minute time intervals for a period of 3 months (August-October 2010) for a total of over 5500 data points at a single spring. Salinity is plotted against pH (a), and grouped according to the number of data points occurring in a given salinity range (b). As depicted, 93% of data points fall above a salinity of 30, and salinity never drops below 27 at the center of discharge. The lower salinity conditions are during low tide in the rainy season and the conditions do not prevail for more than a one hour. Agreement (1 s.d.) between the pH of the sensor values and the discrete measurements (calculated pH) is approximately ±0.07. (DOC) [file pone.0146707.s001.doc]

Supplementary Material

Fig. S1**: Salinity and pH over time as measured by an autonomous sensor.**

Salinity and pH were measured at 15 minute time intervals for a period of 3 months (August-October 2010) for a total of over 5500 data points at a single spring. Salinity is plotted against pH (**a**), and grouped according to the number of data points occurring in a given salinity range (**b**). As depicted, 93% of data points fall above a salinity of 30, and salinity never drops below 27 at the center of discharge. The lower salinity conditions are during low tide in the rainy season and the conditions do not prevail for more than a one hour. Agreement (1 s.d.) between the pH of the sensor values and the discrete measurements (calculated pH) is approximately ±0.07. A more thorough investigation of water chemistry is provided by Paytan et al. (2014) [26].
